# Biogenic Selenium Nanoparticles Alleviate Intestinal Epithelial Barrier Damage through Regulating Endoplasmic Reticulum Stress-Mediated Mitophagy

**DOI:** 10.1155/2022/3982613

**Published:** 2022-08-05

**Authors:** Lei Qiao, Shuqi Yan, Xina Dou, Xiaofan Song, Jiajing Chang, Shanyao Pi, Xinyi Zhang, Chunlan Xu

**Affiliations:** The Key Laboratory for Space Bioscience and Biotechnology, School of Life Sciences, Northwestern Polytechnical University, Xi'an, Shaanxi 710072, China

## Abstract

The intestinal barrier plays a fundamental role in body health. Intracellular redox imbalance can trigger endoplasmic reticulum stress (ERS) and mitophagy, leading to intestinal barrier damage. Our previous studies demonstrated that mitophagy is closely associated with the protective effects of biogenic selenium nanoparticles (SeNPs) on intestinal epithelial barrier function. Thus, we hypothesize that ERS and mitophagy are likely involved in the regulatory effects of SeNPs on oxidative stress-induced intestinal epithelial barrier dysfunction. The results showed that oxidative stress or ERS caused the increase of intestinal epithelial permeability. SeNPs effectively alleviated hydrogen peroxide (H_2_O_2_-)-induced structural damage of endoplasmic reticulum (ER) and mitochondria of porcine jejunal epithelial cells (IPEC-J2). SeNPs significantly decreased intracellular inositol triphosphate (IP3) and Ca^2+^ concentration, down-regulated inositol trisphosphate receptor (IP3R) expression level, and up-regulated ER-resident selenoproteins mRNA levels in IPEC-J2 cells exposed to H_2_O_2_. In addition, SeNPs pretreatment significantly decreased the intracellular Ca^2+^, IP3, IP3R, and reactive oxygen species (ROS) levels; protected the structure and function of ER and mitochondria; and effectively alleviated the increase of intestinal epithelial permeability of IPEC-J2 cells exposed to tunicamycin (TM). Moreover, SeNPs significantly inhibited the colocalization of mitochondria and lysosomes. Furthermore, compared with TM model group, SeNPs significantly inhibited the activation of PERK/eIF2*α*/ATF4 and AMPK/mTOR/PINK1 signaling pathway. The PERK agonist (CCT020312) and the AMPK agonist (AICAR) could reverse the protective effects of SeNPs on IPEC-J2 cells. The PERK inhibitor (GSK2656157) and the AMPK inhibitor (compound C) had a similar effect on IPEC-J2 cells as that of SeNPs. In summary, the protective effects of SeNPs on intestinal barrier dysfunction are closely associated with ERS-related PERK and mitophagy-related AMPK signaling pathway.

## 1. Introduction

The intestinal epithelium acts as a barrier between the contents of the intestinal lumen and the underlying immune system while maintaining the transport of water, nutrients, and ions in the body. Impaired intestinal epithelial barrier function is associated with a variety of gastrointestinal and systemic diseases, including inflammatory bowel disease (IBD). The endoplasmic reticulum (ER), as one of the most important organelles in cells, is the main site of protein synthesis, folding, lipid synthesis, and calcium storage [1, 2]. Intestinal epithelial cells (IECs) have a well-developed ER structure and are one of the most metabolically vigorous cell types. Sustained and excessive endoplasmic reticulum stress (ERS) can induce autophagy through the unfolded protein response (UPR) in IECs, thereby eliciting an inflammatory response. Excessive ERS can also disrupt the intestinal epithelial barrier and ultimately lead to ulcerative colitis (UC) [3–5]. Under the action of various stressors, ER dysfunction, imbalance of calcium homeostasis, and accumulation of misfolded or unfolded proteins in the ER lumen, cells undergo an ERS. When the stressor is too strong or lasts too long, unfolded/misfolded protein accumulates in the ER, and ERS initiates the corresponding signal transduction pathway to induce cell apoptosis [6]. ERS can induce intestinal inflammation, leading to the destruction of the intestinal mucosal mechanical barrier, which in turn induces a variety of diseases [7, 8]. ERS is involved in the homeostasis of IECs and can induce IBD.

Autophagy is a lysosomal degradation pathway, which is essential for survival, differentiation, development, and homeostasis, and has the effect of protecting organisms from various diseases [9, 10]. Mitophagy was identified as a key protective mechanism during ERS [11, 12]. The interplay of mitophagy and ERS is largely considered as a potential mechanism for the epithelial barrier breakdown in IBD [13, 14]. Autophagy is widespread in eukaryotic cells, and moderate autophagy can maintain cell homeostasis [15]; however, it is also believed that overactivated autophagy can induce cell apoptosis and cause cell damage [16]. Regulation of cellular stress by autophagy and protection from stress-induced apoptosis is one aspect of how autophagy plays a role in IEC death [17, 18]. Autophagy has also been implicated in modulating tight junctions (TJs) to support IEC-dendritic cell interactions during luminal antigen sampling and to prevent changes in barrier permeability [19]. Autophagy mainly removes some long-lived proteins, protein aggregates, and dysfunctional organelles, including ER, mitochondria, and ribosomes [20, 21]. IECs require autophagy to manage reactive oxygen species (ROS-)-induced oxidative stress [22–24]. The overproduction of ROS in mitochondria can cause mitochondrial DNA mutations and dysfunction [25]. As a selective autophagy method, mitochondrial autophagy is an indispensable part of the mitochondrial quality control process [26].

The ER and mitochondria are closely related important organelles in intestinal epithelial cells. The redox imbalance in the ER can trigger the occurrence of ERS and the generation of ROS, and mitochondria are the main sites of ROS and ATP generation. The proximity of mitochondria to the ER ensures the ATP demand of the ER. Therefore, mitochondrial homeostasis plays an important role in improving ERS and maintaining ER homeostasis. ERS may synergize with mitochondrial biosynthesis to regulate the reestablishment of cellular homeostasis. Our previous studies have demonstrated that SeNPs can effectively alleviate intestinal epithelial barrier dysfunction and mitochondrial dysfunction caused by oxidative stress. Pretreatment with SeNPs caused the down-regulation of intracellular mitophagy-related protein expression levels in cells exposed to hydrogen peroxide (H_2_O_2_) [27]. ERS can alleviate mitochondrial stress-induced intestinal epithelial barrier dysfunction. However, the relationship of mitophagy-mediated protective effects of SeNPs on oxidative stress-induced intestinal epithelial barrier dysfunction with ERS remains unclear. Thus, we hypothesize that ERS and mitophagy are likely involved in the regulatory effects of SeNPs on oxidative stress-induced intestinal epithelial barrier dysfunction.

Therefore, this study was conducted to investigate the role of ERS in the protective effects of biogenic SeNPs on intestinal epithelial barrier injury and its relationship with mitophagy through the establishment of H_2_O_2_-induced oxidative stress model in porcine jejunal epithelial cells (IPEC-J2). Subsequently, the ERS model was further established using tunicamycin (TM) to study the effect of ERS on mitochondrial function and intestinal epithelial cell permeability, as well as the regulatory effect of SeNPs and its effects on ERS-related protein kinase R-like endoplasmic reticulum kinase (PERK) and mitophagy-related AMP-activated kinase (AMPK) signaling pathway. Moreover, the PERK agonist CCT020312, the PERK inhibitor GSK2656157, the AMPK agonist AICAR, and the AMPK inhibitor Compound C interference experiments were used to verify the role of PERK and AMPK-mediated signaling pathway in the protective effects of SeNPs on intestinal epithelial barrier injury.

## 2. Material and Methods

### 2.1. Reagents

SeNPs were synthesized by *Lactobacillus casei* ATCC 393 (*L. casei* ATCC 393) according to our previously established methods [28]. TM was obtained from Shanghai Yuanye Bio-Technology (Shanghai, China). Sodium selenite, FITC-Dextran (4 kDa), and H_2_O_2_ were purchased from Sigma-Aldrich (St. Louis, MO). Dulbecco's Modified Eagle Medium, penicillin/streptomycin, 0.25% trypsin-EDTA, and fetal bovine serum (FBS) were purchased from Biological Industries. ELISA kits for inositol triphosphate (IP3) and 8-OHdG were purchased from Jianglaibio Company (Shanghai, China). Mitochondrial membrane potential (MMP) assay kit, ROS detection kit and ATP assay kit, mitochondrial permeability transition pore (MPTP) assay kit, lysosome detection kit, cell counting kit-8 (CCK-8), and Fluo-4AM were purchased from Beyotime Biotechnology (Shanghai, China). Glutathione peroxidase (GPx), malondialdehyde (MDA), total superoxide dismutase (T-SOD), thioredoxin reductase (TrxR), total antioxidant capacity (T-AOC) assay kits, 4,6-diamidino-2-phenylindole (DAPI), and bicinchoninic acid (BCA) protein assay kit were purchased from Solarbio Life Science (Beijing, China). TRIzol reagent and reagents for cell culture were purchased from Gibco-Invitrogen. Primary antibodies against occludin, claudin-1, inositol 1,4,5-triphate receptor (IP3R), PERK, p-PERK activating transcription factor 4 (ATF4), eukaryotic initiation factor 2 (eIF2*α*), p-eIF2*α*, glucose-regulated protein 78 (GRP 78), AMPK, p-AMPK, microtubule-associated protein light chain 3 (LC3B), mechanistic target of rapamycin (mTOR), p-mTOR (Ser2448), unc-51 like autophagy activating kinase 1 (ULK 1), and corresponding secondary antibody were purchased from ABclonal Technology Co. (Wuhan, China). The PERK agonist CCT020312, the PERK inhibitor GSK2656157, the AMPK agonist AICAR, and the AMPK inhibitor Compound C were purchased from MedChemExpress (Monmouth Junction, USA).

### 2.2. IPEC-J2 Cell Culture and Drug Intervention

IPEC-J2 cells were donated by Professor Yizhen Wang's research group from the School of Animal Science, Zhejiang University. IPEC-J2 cells were grown in Dulbecco's Modified Eagle medium with 10% FBS and 1% antibiotic mixture (100 U/mL of penicillin and 100 *μ*g/mL streptomycin) at 37°C in a 5% CO_2_ atmosphere.

For investigating the effects of ERS-mediated mitophagy in SeNPs protecting IPEC-J2 from oxidative stress, H_2_O_2_/TM-induced intestinal epithelial barrier damage model of IPEC-J2 cells was established, respectively. (1) In the experiment of SeNPs protecting IPEC-J2 from oxidative stress damage, the experimental groups were as follows: the normal control group, the H_2_O_2_ model group, the SeNPs + H_2_O_2_ group, and the SeNPs group. IPEC-J2 cells were seeded at the density of 1 × 10^5^ cell/mL in 100-mm cell culture dish. After overnight culture, cells from SeNPs pretreatment group were cultured with SeNPs solution containing 8 *μ*g Se/mL for 12 h, and the other groups were added with an equal volume of FBS-free medium. Subsequently, cells from the H_2_O_2_ oxidative stress groups were exposed to 500 *μ*M H_2_O_2_ and cultured for 12 h. (2) In the experiment of SeNPs protecting IPEC-J2 from ERS damage, the groups included were as follows: the normal control group, the TM model group, the SeNPs +TM group, and the SeNPs group. Cells from SeNPs pretreatment group were cultivated with SeNPs solution containing 8 *μ*g Se/mL for 12 h. Other groups were added an equal volume of FBS-free medium. Then, TM model group and SeNPs protective group were exposed to 10 *μ*g/mL TM for 18 h.

### 2.3. Ultrastructure of ER and Mitochondria

After the above treatments, the cells were digested with trypsin containing EDTA, and collected into a 10 mL centrifuge tube. After centrifugation at 1,000 rpm for 10 min, the supernatant was discarded. Then, the cells were resuspended in PBS and centrifuged at 1,500 rpm for 10 min at 4°C. Then, the cells were fixed in 2.5% glutaraldehyde and placed in a refrigerator at 4°C overnight. After samples were processed according to the standard procedures, including staining, dehydration, embedding, and slicing into ultra-thin sections, the ultrastructure of mitochondria was observed on a Hitachi HT7800 transmission electron microscope (Hitachi Ltd., Tokyo, Japan).

### 2.4. mRNA Expression Levels of ER-Resident Selenoproteins Analysis

IPEC-J2 cells were grouped and treated as described above. After the above treatments, total RNA was extracted from cells using the RNAex pro reagent. The quality and concentration of the total RNA were measured with an Implen nanophotometer (Munich, Germany). Then, cDNA was synthesized using an Evo M-MLV mix kit with gDNA clean. Subsequently, real-time PCR was performed on a CFX96 Touch™ Real-Time PCR Detection System (Bio-Rad, USA) according to the SYBR® green premix pro Taq HS qPCR kit. The primers for target genes and a housekeeping gene (*β*-actin) are listed in Table 1. The relative mRNA abundance of the selected genes was normalized to *β*-actin expression and was then calculated using the 2^−*ΔΔ*Ct^ method.

### 2.5. Detection of Ca^2+^ Concentration in the Cytoplasm

Cells were grouped and treated as described above. After treatments, the culture medium was discarded. Cells were washed three times with HBSS and then cocultivated with Fluo-4AM for 30 min. Finally, calcium ion concentration in the cytoplasm was detected by an inverted fluorescence microscope (Leica DMIL, Germany).

### 2.6. Calcium Channel Protein Content Detection

Cells were grouped and treated as described above. After treatments, the total protein was extracted from the cells, and the protein concentration was determined using the BCA protein assay kit. Finally, the intracellular IP3 content of each experimental group was measured by IP3 ELISA kit according to the manufacturer's instructions.

### 2.7. CCK8 Assay

First, IPEC-J2 cells were seeded into 96-well plates (Corning, NY, USA) at a concentration of 5 × 10^3^ cells per well overnight to allow cell attachment. The cells were treated with TM at different doses (0, 2, 4, 6, 8, 10, and 12 *μ*M) for 18 h. Then, 10 *μ*L CCK-8 was added to each well and incubated with cells for 2 h. The optical density was detected at 570 nm using a microplate reader (Bio-Rad, Hercules, CA, USA).

### 2.8. Intestinal Epithelial Permeability Analysis

IPEC-J2 cells were seeded at a concentration of 1 × 10^5^ cells/per well in 24-well transwell plate with a pore size of 0.4 *μ*m (Corning, NY, USA) and cultured at 37°C, 5% CO_2_. When the transepithelial electrical resistance (TEER) was stable (approximately 150 *Ω* cm^2^), they were ready to be studied. Cells were grouped and treated as described above. The TEER values were measured using a Millicell resistance system (Merck Millipore, Darmstadt, Germany) before SeNPs pretreatment (prior treatment), after SeNPs pretreatment (posttreatment), and after H_2_O_2_ treatment (oxidative stress), respectively. Immediately after the last determination of TEER value, 100 *μ*L of FITC-dextran (2.2 mg/mL) was added to the transwell inserts (upper compartments). The plates were cultured at 37°C for 30 min, and then, 100 *μ*L of medium from each plate well (lower compartments) was collected to detect the fluorescence intensity at an excitation wavelength of 480 nm and an emission wavelength of 520 nm.

### 2.9. Detection of ROS Generation

Cells were grouped and treated as described above. After treatments, cells were incubated with 10 *μ*M 2'-7'dichlorofluorescin diacetate (DCFH-DA) at 37°C for 20 min and then washed three times with FBS-free medium. Finally, the fluorescence intensity was detected under an inverted fluorescence microscope (Leica DMIL, Germany).

### 2.10. Antioxidant Capacity Evaluation

Cells are grouped and treated as described above. After treatments, intracellular MDA content and the activities of GPx, T-AOC, T-SOD, and TrxR were detected by corresponding kits according to the manufacturer's instructions.

### 2.11. ATP Level

Cells were groups and treated as described above. After the treatments, the level of ATP was determined by the corresponding kit according to the manufacturer's instructions.

### 2.12. 8-OHdG Level

Cells were grouped and treated as described above. After treatments, the intracellular 8-OHdG content of each experimental group was measured by 8-OHdG ELISA kit according to the manufacturer's instructions.

### 2.13. MMP (*Δψ*m) Assay

MMP of experimental cells was detected by MMP assay kit with JC-1 staining. Cell treatment methods were the same as described above. After that, cells were washed three times with PBS and stained with JC-1 for 20 min. Then, cells were washed three times with JC-1 buffer after JC-1 solution was discarded. Finally, images were captured with a laser scanning confocal microscope (Leica sp5, Leica Microsystems). Green monomeric JC-1 and red aggregated JC-1 were detected at an emission wavelength of 530 nm and 590 nm, respectively.

### 2.14. MPTP Opening Detection

Cell treatment methods were the same as described above. Then, the cells were incubated with 2 *μ*M calcein for 30 min in the absence of light. The cells were subsequently washed twice with PBS and then exposed to 2 mM CoCl_2_ for 15 min. The calcein fluorescence is compartmentalized within the mitochondria until the MPTP opening permits the distribution of cobalt inside the mitochondria, which results in the quenching of calcein fluorescence in the mitochondrial matrix. After three washes with PBS, fluorescence imaging of cells was performed with excitation at 488 nm and emission at 520 nm using the laser scanning confocal microscope (Leica sp5, Leica Microsystems).

### 2.15. Colocalization of Mitochondria and Lysosomes

Cells were groups and treated as described above. After treatments, the cells were cocultivated with Mito-Tracker Green fluorescent probe for 45 min at 37°C, and 5% CO_2_, and the cells were washed twice with PBS. Then, cells were cocultivated with Lyso-Tracker Red fluorescent probe for 45 min at 37°C and 5% CO_2_ and washed twice with PBS. Finally, mitochondria with green fluorescence and lysosomes with red fluorescence were observed under confocal laser scanning microscope (Leica sp5, Leica Microsystems).

### 2.16. Western Blot Analysis

The total protein was isolated from cells using the RIPA buffer containing protease and phosphatase inhibitor cocktail. The protein concentration of the samples was measured by the BCA protein assay kit. Before loading, protein samples were boiled in the loading buffer, and then, samples of equal volume with equal amount of protein were loaded onto the sodium dodecyl sulfate–polyacrylamide gel electrophoresis (SDS-PAGE) gel and then transferred to the PVDF membrane. The membranes were incubated overnight at 4°C with the appropriate primary antibodies for occludin, claudin-1, IP3R, PERK, p-PERK, ATF4, eIF2*α*, p-eIF2*α*, Grp 78, AMPK, p-AMPK, LC3B, mTOR, p-mTOR (Ser2448), ULK 1, and *β*-actin followed by incubation for 1 h at room temperature with the appropriate secondary antibodies. Immunoreactive protein bands were visualized with the clarity Western ECL substrate kit (BioRad, USA) using Tanon 5200 Multi (Shanghai, China) and quantified using the Image J analyzer software (National Institute of Health, Bethesda, MD, USA). All blots or gels derive from the same experiment and that they were processed in parallel.

### 2.17. Effect of PERK Agonist and Inhibitor on the Permeability and Oxidative Stress of IPEC-J2 Cells Exposed to H_2_O_2_

To investigate the role of PERK signaling in SeNPs protecting IPEC-J2 from oxidative stress, we first exposed cells to the SeNPs, the PERK agonist CCT020312 (10 *μ*M, dissolved in DMSO), and the PERK inhibitor GSK2656157 (1 *μ*M, dissolved in DMSO) for 12 h, followed by the addition of H_2_O_2_ (500 *μ*M) for 12 h to establish an oxidative stress model. The groups included were as follows: normal control group, H_2_O_2_ group, SeNPs + H_2_O_2_ group, SeNPs group, CCT020312 + SeNPs + H_2_O_2_ group, and GSK2656157 + H_2_O_2_ group. After the above treatments, Western blot was used to detect the ERS-related PERK signaling pathway and mitophagy-related AMPK signaling pathway and the expression levels of TJs proteins. Moreover, the content of intracellular ROS was also detected by corresponding kit.

### 2.18. Effect of AMPK Agonist and Inhibitor on the Permeability and Oxidative Stress of IPEC-J2 Cells Exposed to H_2_O_2_

To investigate the role of AMPK signaling in SeNPs protecting IPEC-J2 from oxidative stress, we first exposed cells to the SeNPs, the AMPK agonist AICAR (1 mM, dissolved in DMSO), and the AMPK inhibitor compound C (10 *μ*M, dissolved in DMSO) for 12 h, followed by the addition of H_2_O_2_ (500 *μ*M) for 12 h to establish an oxidative stress model. The groups included were as follows: normal control group, H_2_O_2_ group, SeNPs + H_2_O_2_ group, SeNPs group, AICAR + SeNPs + H_2_O_2_ group, and compound C + H_2_O_2_ group. After the above treatments, Western blot was used to detect the mitophagy-related AMPK signaling pathway and the expression of TJs. Moreover, the content of intracellular ROS was also detected by corresponding kit.

### 2.19. Statistical Analysis

All of the experimental data were performed by GraphPad Prism 5 statistical software (Graph-Pad Software Inc., San Diego, CA, USA) and expressed as mean ± standard error of mean (SEM). Data were analyzed statistically by one-way analysis of variance (ANOVA) or Student's *t*-test. Probability values <0.05 were considered statistically significant. All determinations were performed in at least three independent experiments.

## 3. Results

### 3.1. Effects of SeNPs on the Ultrastructure of ER and Mitochondria and the mRNA Levels of ER-Resident Selenoproteins in IPEC-J2 Cells Exposed to H_2_O_2_

**Figure 1:**
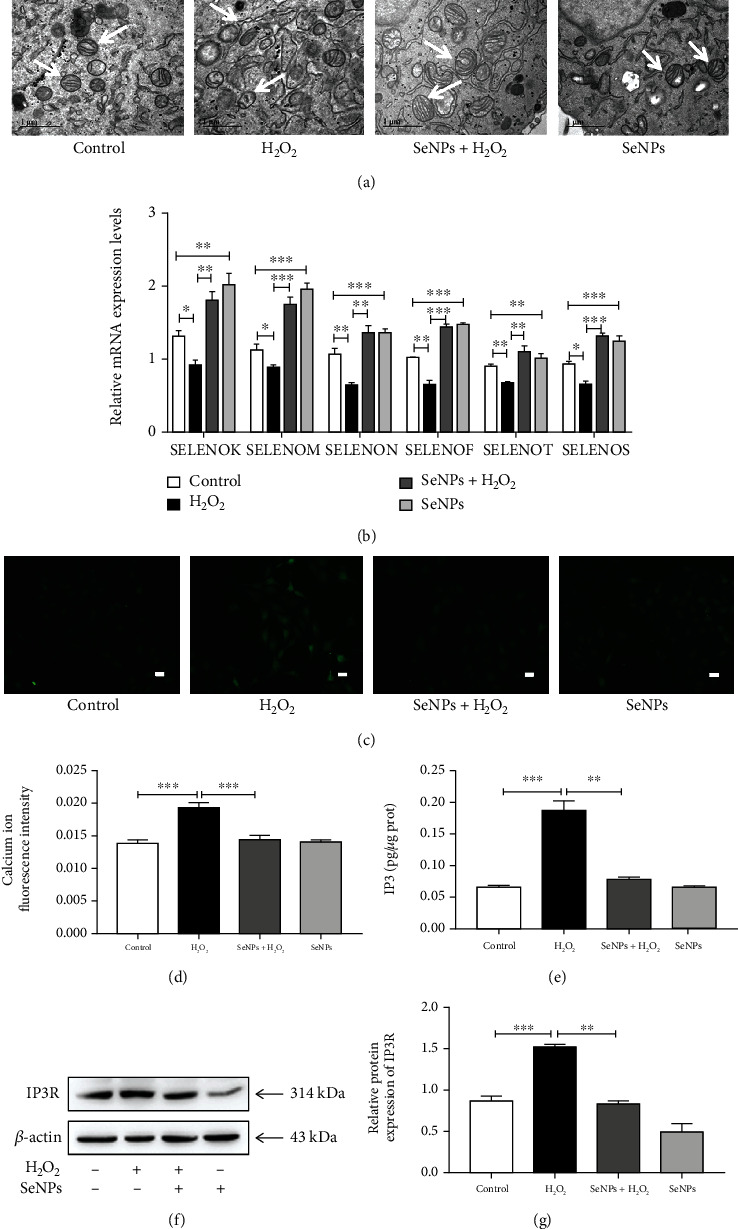
Effects of SeNPs on ER structure and function of IPECJ2 cells exposed to H_2_O_2_. (a) Protective effect of SeNPs on the ultrastructure of ER and mitochondria in IPEC-J2 cells exposed to H_2_O_2_. (b) Regulatory effect of SeNPs on the mRNA levels of ER-resident selenoproteins. (c) Intracellular Ca^2+^ concentration was detected by Fluo-4 AM staining (scale bar: 100 *μ*M). (d) Quantitative analysis of intracellular Ca^2+^ concentration. (e) Intracellular IP3 content. (f) The expression level of IP3R was detected by Western blot analysis. (g) Quantified statistical result of IP3R expression level. All data are presented as the mean ± SEM of three independent experiments. ^∗^*P* < 0.05, ^∗∗^*P* < 0.01, and ^∗∗∗^*P* < 0.001. SeNPs: selenium nanoparticles; ER: endoplasmic reticulum; H_2_O_2_: hydrogen peroxide; IP3: inositol triphosphate; IP3R: inositol trisphosphate receptor.

As shown in Figure 1(a), the ER in the control group and the SeNPs-protective group is flat and sac-like and arranged neatly; mitochondria were columnar or reticular, with clear mitochondrial cristae, normal matrix density, and intact mitochondrial membranes. However, ER and mitochondrial structures were significantly damaged in the H_2_O_2_ model group. The ER was vacuolated and swollen, mitochondrial cristae were blurred, matrix density was low, and some mitochondrial membranes were incomplete. In addition, SeNPs pretreatment effectively alleviated H_2_O_2_-induced ER and mitochondria structural damage. Compared with the normal control group, the mRNA levels of ER-resident selenoproteins including SELENOK, SELENOM, SELENON, SELENOF, SELENOT, and SELENOS were significantly decreased in the cells from the H_2_O_2_ model group. However, compared with the H_2_O_2_ model group, the mRNA levels of SELENOK, SELENOM, SELENON, SELENOF, SELENOT, and SELENOS were significantly increased in the cells from the SeNPs-protective group. In addition, compared with the normal control group, the SeNPs-treated group significantly increased the mRNA levels of SELENOK, SELENOM, SELENON, SELENOF, SELENOT, and SELENOS (Figure 1(b)).

### 3.2. Regulatory Effects of SeNPs on Intracellular Calcium Homeostasis in IPEC-J2 Cells Exposed to H_2_O_2_

As shown in Figures 1(c) and 1(d), compared with the normal control group, the Ca^2+^ concentration in the H_2_O_2_ model group is significantly increased. Compared with the H_2_O_2_ model group, SeNPs pretreatment significantly inhibited H_2_O_2_-induced increase of Ca^2+^ concentration in the cytoplasm of IPEC-J2 cells. As shown in Figure 1(e), compared with the normal control group, the IP3 content was significantly increased in the H_2_O_2_ model group. However, compared with the H_2_O_2_ model group, SeNPs pretreatment significantly reduced the H_2_O_2_-induced increase in IP3 content and down-regulated the expression level of IP3R (Figures 1(f) and 1(g)).

### 3.3. Regulatory Effects of SeNPs on the ER Stress-Related PERK Signaling Pathway in IPEC-J2 Cells Induced by H_2_O_2_

**Figure 2:**
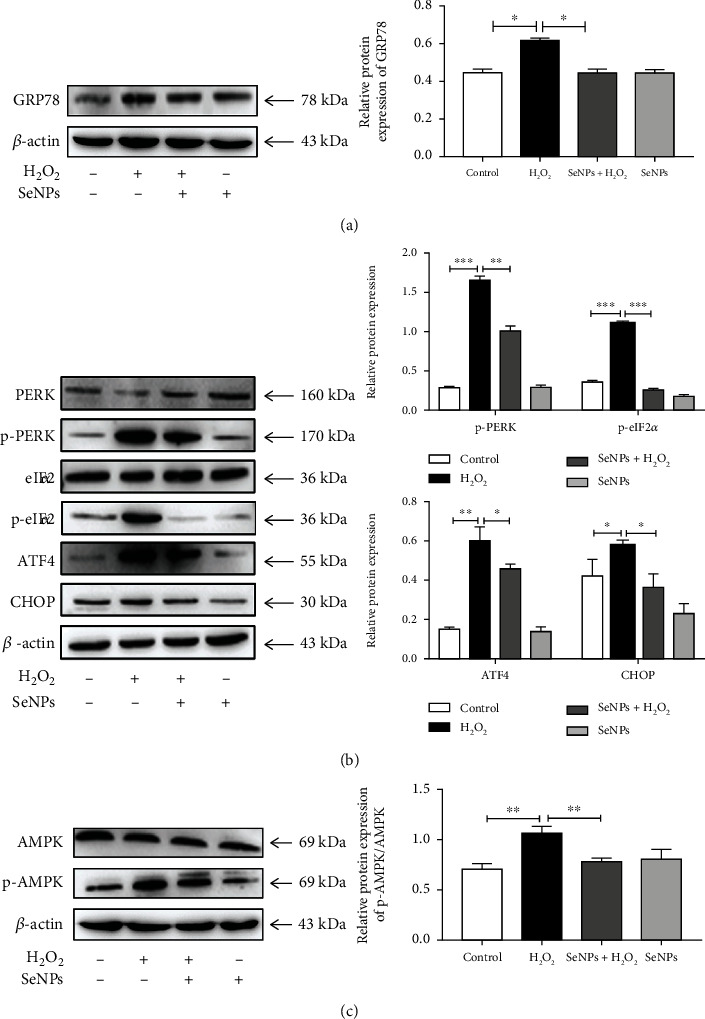
Regulatory effects of SeNPs on PERK and AMPK signaling pathway in IPEC-J2 cells exposed to H_2_O_2_. (a) The expression level of GRP78 was detected by Western blot analysis. (b) The expression levels of PERK, p-PERK, eIF2*α*, p-eIF2*α*, ATF4, and CHOP were detected by Western blot analysis. (c) The expression level of p-AMPK/AMPK was detected by Western blot analysis. All data are presented as the mean ± SEM of three independent experiments. ^∗^*P* < 0.05, ^∗∗^*P* < 0.01, and ^∗∗∗^*P* < 0.001. SeNPs: selenium nanoparticles; H_2_O_2_: hydrogen peroxide; XBP1: X-box binding protein 1; GRP78: glucose-regulated protein 78; PERK: protein kinase R-like endoplasmic reticulum kinase; eIF2*α*: eukaryotic initiation factor 2; ATF4: activating transcription factor 4; AMPK: AMP-activated kinase.

As shown in Figures 2(a) and 2(b), compared with the normal control group, the expression levels of GRP78, p-PERK, p-eIIF2*α*, ATF4, and CHOP are significantly increased in the H_2_O_2_ model group. Compared with the H_2_O_2_ model group, SeNPs pretreatment significantly down-regulated the expression levels of GRP78, p-PERK, p-eIIF2*α*, ATF4, and CHOP. Meanwhile, compared with the H_2_O_2_ model group, SeNPs pretreatment significantly down-regulated the expression level of p-AMPK/AMPK. However, compared with the normal control group, the SeNPs-treated group significantly increased the protein expression level of p-AMPK/AMPK (Figure 2(c)).

### 3.4. Protective Effect of SeNPs on the Intestinal Epithelial Permeability of IPEC-J2 Cells Exposed to TM

**Figure 3:**
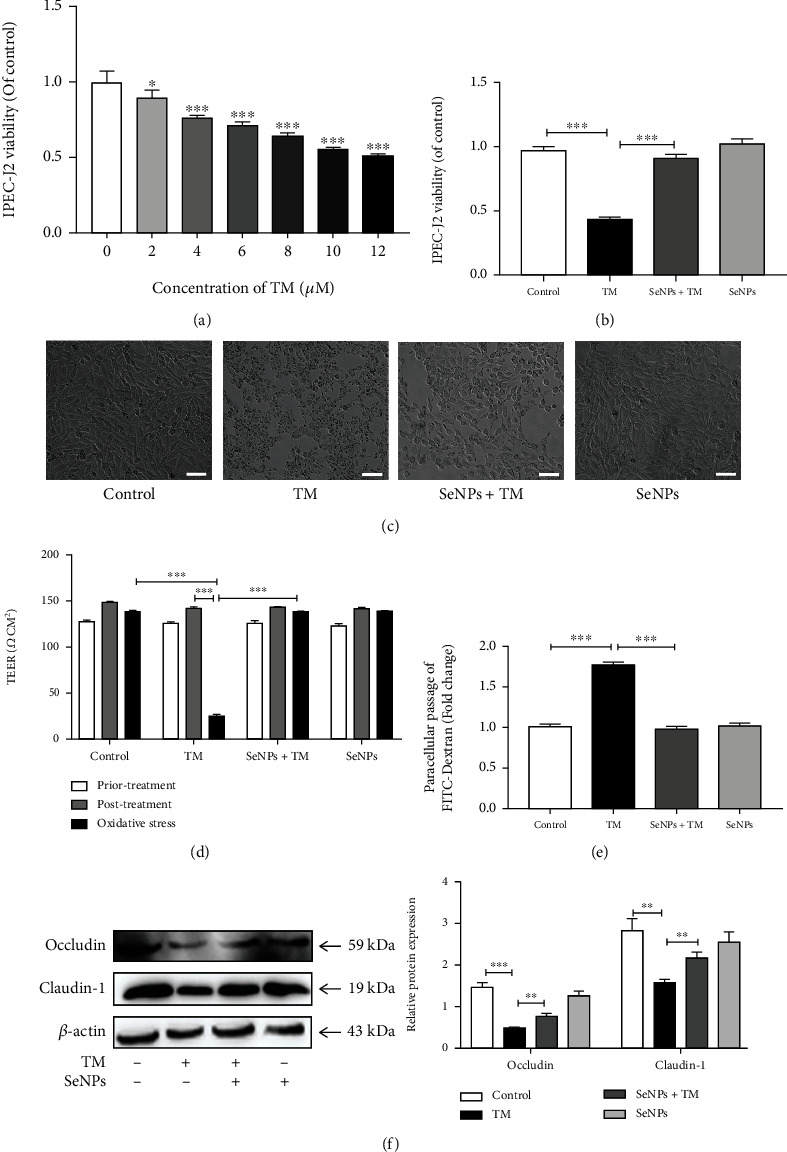
Regulatory effects of SeNPs on TM-induced ERS in IPEC-J2 cells. (a) Effect of different concentrations of TM on the viability of IPEC-J2 cells. (b) Effect of SeNPs on the viability of IPEC-J2 cells exposed to TM. (c) Effect of SeNPs the cell morphology of IPEC-J2 cells (scale bar: 50 *μ*M). (d) Effect of SeNPs on the TEER value of IPEC-J2 cells exposed to TM. (e) Effect of SeNPs on the FITC-Dextran flux cross IPEC-J2 cells exposed to TM. (f) The expression levels of occludin and claudin-1 were detected by Western blot analysis. All data are presented as the means ± SEM of three independent experiments. ^∗^*P* < 0.05, ^∗∗^*P* < 0.01, and ^∗∗∗^*P* < 0.001. SeNPs: selenium nanoparticles; TM: tunicamycin; TEER: transepithelial electrical resistance.

As shown in Figure 3(a), compared with the normal control group, exposure to TM (2, 4, 6, 8, 10, and 12 *μ*M) significantly decreased cell viability in a dose-dependent manner. Among of them, the cell viability decreased to 55% after exposure to 10 *μ*M TM for 18 h. As shown in Figures 3(b) and 3(c), compared with the TM model group, SeNPs pretreatment significantly suppresses the cytotoxic effect of TM, increases cell survival, and significantly alleviates TM-induced cell death. As shown in Figure 3(d), compared with the normal control group, TM exposure causes the reduction of TEER. However, SeNPs significantly attenuated the reduction of TEER induced by TM. In addition, compared with the normal control group, the FITC-dextran fluxes across cells in the TM model group were significantly increased. In contrast, the SeNPs pretreatment group significantly alleviated the increase in TM-induced FITC-dextran fluxes (Figure 3(e)). As shown in Figure 3(f), exposure to TM results in a significant decrease in the protein expression levels of claudin-1 and occludin in IPEC-J2 cells when compared with the normal control group. Compared with the TM model group, SeNPs pretreatment significantly increased the expression levels of claudin-1 and occludin.

### 3.5. Effects of SeNPs on the Antioxidant Capacity of IPEC-J2 Cells Exposed to TM

**Figure 4:**
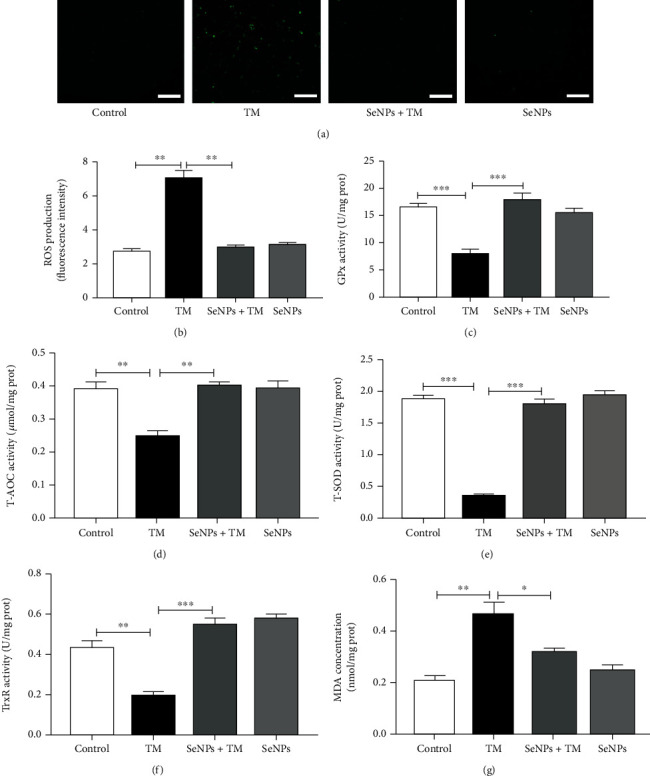
Regulatory effects of the SeNPs on the antioxidant capacity of IPEC-J2 cells exposed to TM. (a) Intracellular ROS level was detected by DCFH-DA fluorescent probe (scale bar: 100 *μ*M). (b) Quantitative analysis of intracellular ROS level. (c) GPx activity. (d) T-AOC activity. (e) T-SOD activity. (f) TrxR activity. (g) The level of MDA. All data are presented as the mean ± SEM of three independent experiments. ^∗^*P* < 0.05, ^∗∗^*P* < 0.01, ^∗∗∗^*P* < 0.001. SeNPs: selenium nanoparticles; TM: tunicamycin; ROS: reactive oxygen species; GPx: glutathione peroxidase; T-AOC: total antioxidant capacity; T-SOD: total superoxide dismutase; TrxR: thioredoxin reductase; MDA: malondialdehyde; MFI: mean fluorescence intensity.

As shown in Figures 4(a) and 4(b), compared with the normal control group, the ROS level in IPEC-J2 cells from the TM model group is significantly increased. However, SeNPs protection significantly inhibited the overproduction of ROS compared with the TM model group. Compared with the normal control group, exposure to TM resulted in a significant decrease in the activities of GPx, T-AOC, T-SOD, and TrxR, and a significant increase in the content of MDA in IPEC-J2 cells. However, SeNPs protection significantly increased the activities of GPx, T-AOC, T-SOD, and TrxR, and significantly decreased the MDA content in IPEC-J2 cells when compared with the TM model group (Figures 4(c)–4(g)).

### 3.6. Effects of SeNPs on the Ultrastructure of ER and Mitochondria and the mRNA Levels of ER-Resident Selenoproteins in IPEC-J2 Cells Exposed to TM

**Figure 5:**
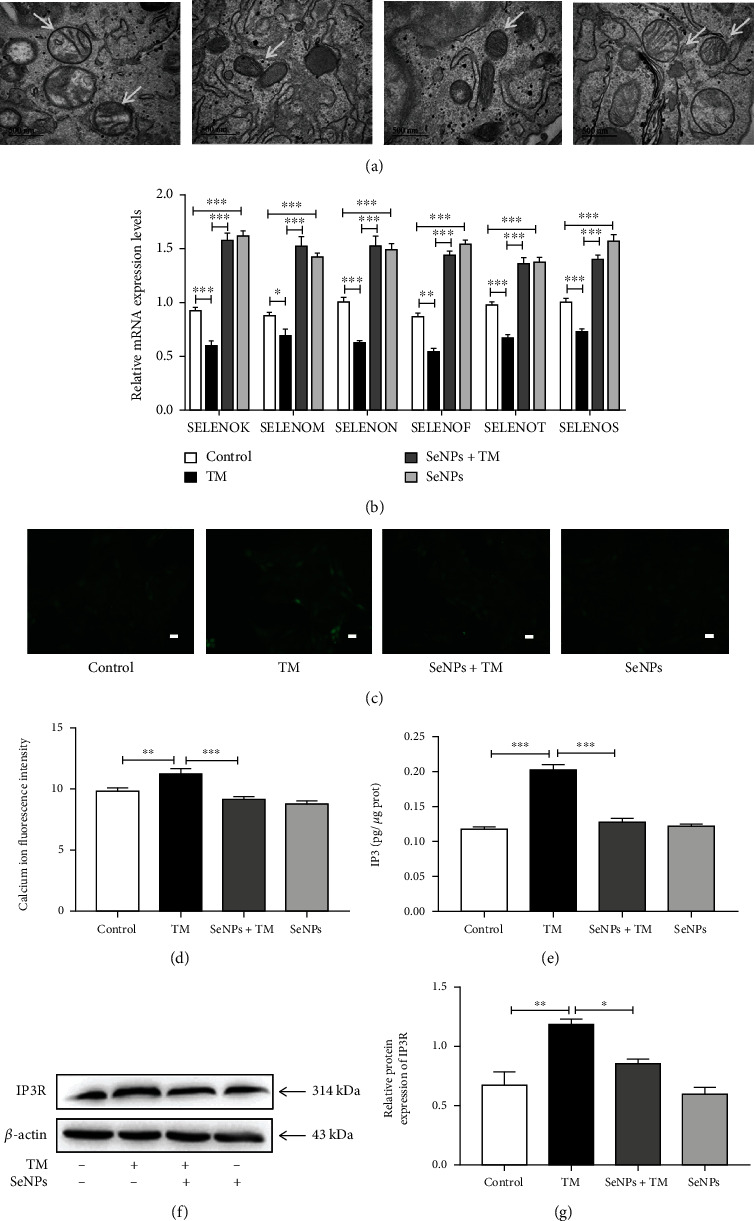
Effects of SeNPs on the structure and function of ER in IPEC-J2 cells exposed to TM. (a) Protective effect of SeNPs on the ultrastructure of ER and mitochondria in IPEC-J2 cells exposed to TM. (b) Regulatory effects of SeNPs on the mRNA levels of ER-resident selenoproteins in IPEC-J2 cells exposed to TM. (c) Intracellular Ca^2+^concentration was detected by Fluo-4 AM fluorescence staining (scale bar: 100 *μ*M). (d) Quantitative analysis of intracellular Ca^2+^ concentration. (e) IP3 content. (f) The expression level of IP3R was detected by Western blot analysis. (g) Quantified statistical results of IP3R expression levels. All data are presented as the mean ± SEM of three independent experiments. ^∗^*P* < 0.05, ^∗∗^*P* < 0.01, and ^∗∗∗^*P* < 0.001. SeNPs: selenium nanoparticles; ER: endoplasmic reticulum; TM: tunicamycin; IP3: inositol triphosphate; IP3R: inositol trisphosphate receptor.

As shown in Figure 5(a), ER is flat and sac-like and neatly arranged in the cells from the normal control group and the SeNPs group. Mitochondria were columnar or reticular, and with clear mitochondrial cristae, normal matrix density, and intact mitochondrial membranes in the cells from the normal control group and the SeNPs group. However, ER and mitochondria were significantly damaged in the TM model group. ER was vacuolated with obvious swelling. Mitochondrial cristae were blurred or even disappeared. The matrix density was low, and some mitochondrial membranes were incomplete. SeNPs pretreatment significantly alleviated TM-induced structural damage of ER and mitochondria. As shown in Figure 5(b), compared with the normal control group, the mRNA levels of SELENOK, SELENOM, SELENON, SELENOF, SELENOT ,and SELENOS in the cells from the TM model group are significantly decreased. However, SeNPs pretreatment significantly alleviated TM-induced decrease in the mRNA levels of SELENOK, SELENOM, SELENON, SELENOF, SELENOT, and SELENOS. Compared with the normal control group, SeNPs significantly increased the mRNA levels of SELENOK, SELENOM, SELENON, SELENOF, SELENOT, and SELENOS.

### 3.7. Regulatory Effect of SeNPs on Intracellular Calcium Homeostasis in IPEC-J2 Cells Exposed to TM

Compared with the normal control group, exposure to TM resulted in a significant increase of cytoplasmic Ca^2+^ concentration. Compared with the TM model group, and SeNPs pretreatment significantly inhibited the increase of cytoplasmic Ca^2+^ concentration in IPEC-J2 cells (Figures 5(c) and 5(d)). As shown in Figure 5(e), compared with the normal control group, the content of IP3 significantly increased in IPEC-J2 cells from the TM model group. Compared with the TM model group, SeNPs protection significantly inhibited TM-induced increase of IP3 content in IPEC-J2 cells. As shown in Figures 5(f) and 5(g), compared with the normal control group, TM exposure results in a significant increase in the protein expression level of IP3R. Compared with the TM model group, the SeNPs pretreatment significantly decreased the protein expression level of IP3R in IPEC-J2 cells exposed to TM.

### 3.8. Regulatory Effects of SeNPs on Mitochondrial Function of IPEC-J2 Cells Exposed to TM

**Figure 6:**
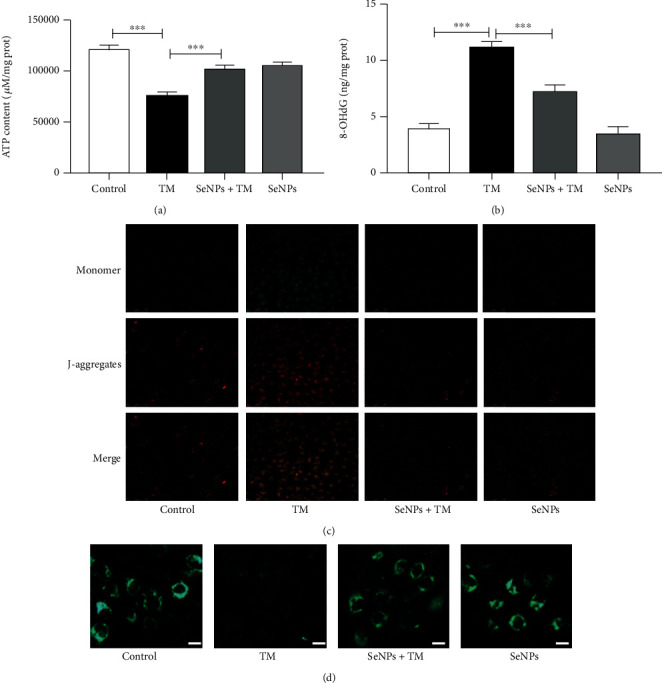
Effects of SeNPs on mitochondrial function of IPEC-J2 cells exposed to TM. (a) ATP level. (b) 8-OHdG level. (c) The MMP was detected by JC-1 staining (scale bar: 75 *μ*M). (d) The mitochondrial permeability transition pore (scale bar: 50 *μ*M). All data are presented as the mean ± SEM of three independent experiments. ^∗^*P* < 0.05 and ^∗∗^*P* < 0.01. SeNPs: selenium nanoparticles; TM: tunicamycin; ATP: mitochondrial adenosine triphosphate; MMP: mitochondrial membrane potential; MPTP: mitochondrial permeability transition pore.

**Figure 7:**
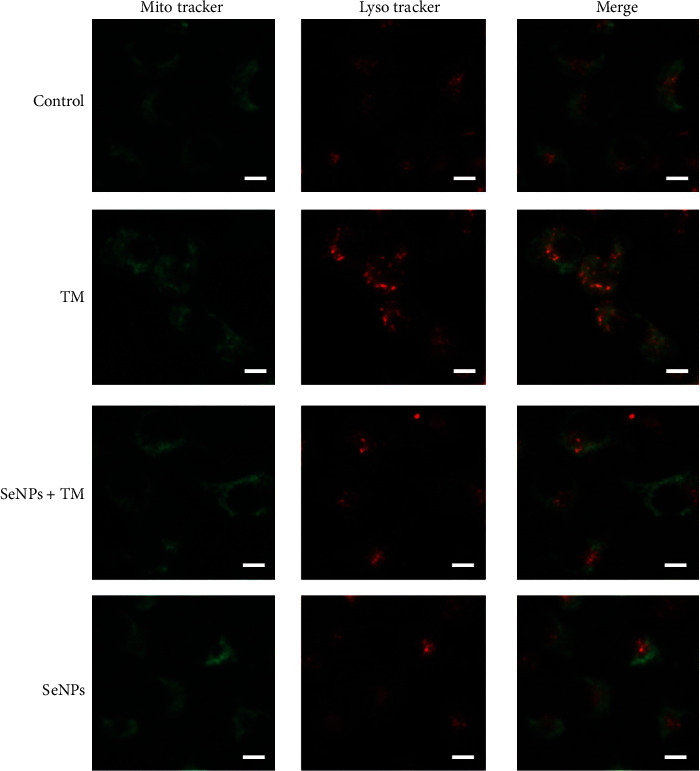
The colocalization of mitochondria and lysosomes in IPEC-J2 cells exposed to TM (Scale bar: 50 *μ*M). All data are presented as the mean ± SEM of three independent experiments. SeNPs: selenium nanoparticles; TM: tunicamycin.

As shown in Figure 6(a), compared with the normal control group, exposure to TM results in a significant decrease of ATP levels. Pretreatment with SeNPs significantly alleviated TM-induced decrease of ATP levels when compared with the TM model group. The level of 8-OHdG (a biomarker of mitochondrial oxidative damage) in the TM model group was significantly higher than that in the normal control group. However, SeNPs administration significantly protects mitochondrial damage from TM-induced ERS (Figure 6(b)). As shown in Figure 6(c), compared with the normal control group, MMP was significantly decreased in the TM model group. However, SeNPs pretreatment significantly inhibited TM-induced reduce of MMP. Furthermore, pretreatment with SeNPs significantly inhibited the TM-induced decrease in MPTP when compared with the TM model group (Figure 6(d)). As shown in Figure 7, in the normal control cells, we observe a clear separation of lysosomes and mitochondria, with red and green fluorescence, respectively. However, in the TM model group, there was an increase in yellow fluorescent spots, which is the colocalization of lysosomes and mitochondria in mitophagy-positive spots (yellow fluorescent spots). In contrast, most of the lysosomes and mitochondria in the cells from SeNPs-pretreated group were isolated. Quantitative analysis results showed that the number of mitophagy-positive spots in the SeNPs-protected group was significantly reduced when compared with the TM model group.

### 3.9. Regulatory Effects of SeNPs on ERS-Mediated Mitophagy Signaling Pathway in IPEC-J2 Cells Exposed to TM

**Figure 8:**
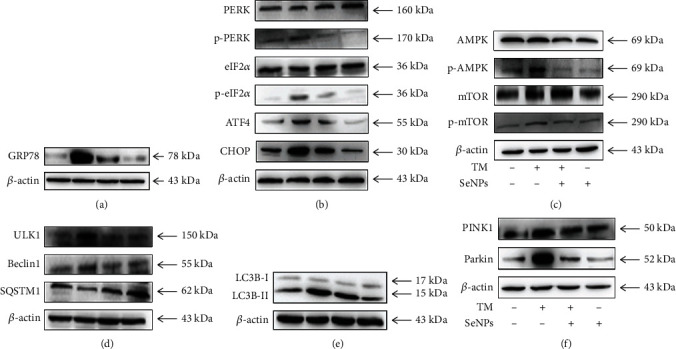
Effects of SeNPs on the PERK and AMPK signaling pathways. (a) The protein expression level of GRP78 was detected by Western blot analysis. (b) The expression levels of PERK signaling pathway were detected by Western blot analysis. (c) The expression levels of p-AMPK, AMPK, mTOR, and p-mTOR were detected by Western blot analysis. (d) The expression levels of ULK1, Beclin1, and SQSTM1 were detected by Western blot analysis. (e) The expression level of LC3B-II/LC3B-I was detected by Western blot analysis. (f) The expression level of PINK1 and Parkin was detected by Western blot analysis. All data are presented as the mean ± SEM of three independent experiments. ^∗^*P* < 0.05 and ^∗∗^*P* < 0.01. SeNPs: selenium nanoparticles; TM: tunicamycin; XBP1: X-box binding protein 1; GRP78: glucose-regulated protein 78; PERK: protein kinase R-like endoplasmic reticulum kinase; eIF2*α*: eukaryotic initiation factor 2; ATF4: activating transcription factor 4; AMPK: AMP-activated kinase; mTOR: mechanistic target of rapamycin, ULK1: unc-51 like autophagy; SQSYM1: sequestosome-1; LC3B: microtubule-associated protein light chain 3; PINK1: PTEN-induced kinase 1.

As shown in Figures 8(a) and 8(b), compared with the normal control group, TM exposure significantly up-regulates the protein expression levels of GRP78, p-PERK, p-eIF2*α*, ATF4, and CHOP. However, compared with the TM model group, SeNPs pretreatment significantly down-regulated the protein expression levels of GRP78, p-PERK, p-eIIF2*α*, ATF4, and CHOP. As shown in Figures 8(c)–8(f), compared with the normal control group, TM exposure significantly increases the protein expression levels of p-AMPK/AMPK, p-mTOR, ULK1, LC3B-II/LC3B-I, PINK1, and Parkin and reduces the protein expression level of SQSTM1. However, compared with the TM model group, SeNPs pretreatment significantly decreased the protein expression levels of p-AMPK/AMPK, p-mTOR, ULK1, LC3B-II/LC3B-I, PINK1, and Parkin and increased the protein expression level of SQSTM1. In addition, compared with the normal control group, SeNPs significantly increased the protein expression levels of p-AMPK/AMPK, Beclin1, LC3B-II/LC3B-I, PINK1, and Parkin.

### 3.10. Effect of PERK Agonist and Inhibitor on ERS-Mediated Mitophagy in IPEC-J2 Cells Exposed to H_2_O_2_

**Figure 9:**
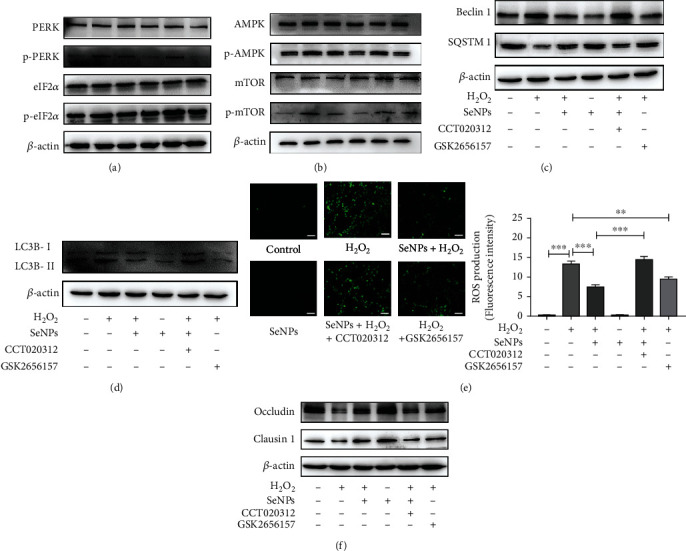
Effect of PERK agonist and inhibitor on ERS-mediated mitophagy in IPEC-J2 cells exposed to H_2_O_2_. (a) The expression levels of PERK signaling pathway-related proteins were detected by Western blot analysis. (b) The expression levels of AMPK, p-AMPK, mTOR, and p-mTOR were detected by Western blot analysis. (c) The expression levels of Beclin1 and SQSTM1 were detected by Western blot analysis. (d) The expression level of LC3B-II/LC3B-I was detected by Western blot analysis. (e) The ROS levels were detected by DCFH-DA fluorescent probe (scale bar: 50 *μ*M). (f) The expression levels of occludin and claudin-1 were detected by Western blot analysis. All data are presented as the mean ± SEM of three independent experiments. ^∗^*P* < 0.05, ^∗∗^*P* < 0.01, and ^∗∗∗^*P* < 0.001. SeNPs: selenium nanoparticles; H_2_O_2_: hydrogen; PERK: protein kinase R-like endoplasmic reticulum kinase; eIF2*α*: eukaryotic initiation factor 2; AMPK: AMP-activated kinase; mTOR: mechanistic target of rapamycin; SQSYM1: sequestosome-1; LC3B: microtubule-associated protein light chain 3.

As shown in Figure 9(a), compared with the normal control group, the expression levels of p-PERK and p-eIF2*α* are significantly increased in cells from the H_2_O_2_ model group, whereas pretreatment with SeNPs significantly decreased the expression levels of p-PERK and p-eIF2*α*. However, compared with SeNPs + H_2_O_2_ group, the expression levels of p-PERK and p-eIF2*α* were more significantly increased in the CCT020312 + SeNPs + H_2_O_2_ group. However, the expression levels of p-PERK and p-eIF2*α* were significantly decreased in the GSK2656157 + H_2_O_2_ group when compared with the H_2_O_2_ model group, which indicated that SeNPs significantly inhibited the activation of PERK signaling pathway. As shown in Figures 9(b)–9(d), compared with the SeNPs + H_2_O_2_ group, PERK agonist CCT020312 significantly increases the expression levels of p-AMPK, p-mTOR, Beclin1, and LC3B-II/LC3B-I, and reduces the expression level of SQSTM1. In addition, SeNPs exhibited a similar effect to that of the inhibitor of PERK (GSK2656157), significantly reducing the high expression of p-AMPK, p-mTOR, Beclin1, and LC3B-II/LC3B-I induced by H_2_O_2_. In order to further verify the protective effect of SeNPs on intestinal barrier oxidative damage, we detected the intracellular ROS content and the expression levels of TJ proteins. As shown in Figures 9(e) and 9(f), compared with the SeNPs + H_2_O_2_ group, CCT020312 + SeNPs + H_2_O_2_ group had a high level of ROS and a low levels of TJ proteins expression. However, GSK2656157 significantly reduced intracellular ROS level and increased TJ proteins levels in the H_2_O_2_ model group. These results suggested that SeNPs effectively alleviated H_2_O_2_-induced intestinal epithelial barrier dysfunction by regulating the ERS-related PERK/eIF2*α*/ATF4-mediated mitophagy signaling pathway.

### 3.11. Effect of AMPK Agonist and Inhibitor on ERS-Mediated Mitophagy in IPEC-J2 Cells Exposed to H_2_O_2_

**Figure 10:**
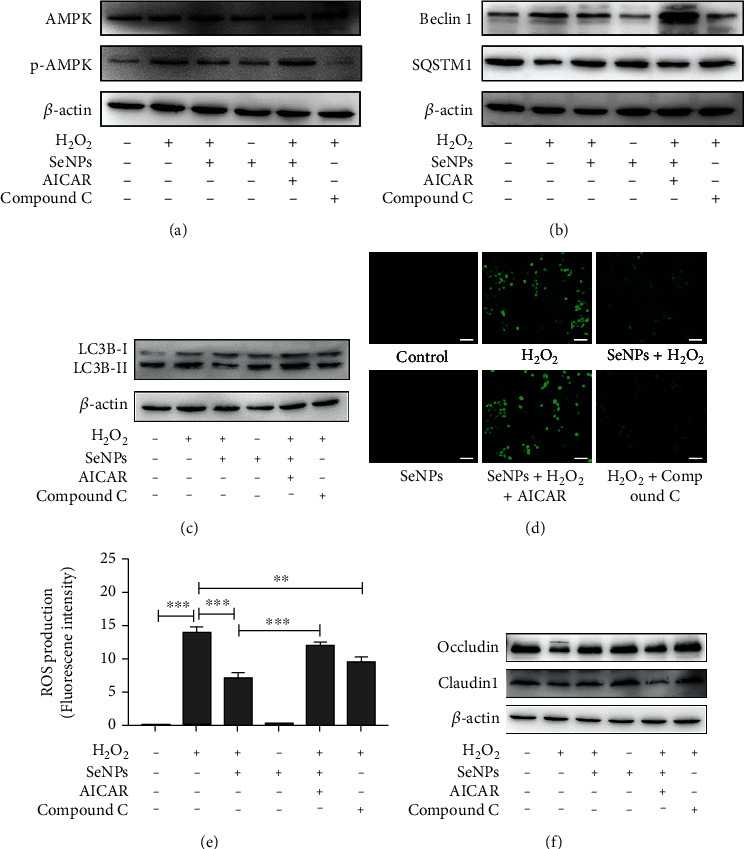
Effect of AMPK agonist and inhibitor on ERS-mediated mitophagy in IPEC-J2 cells exposed to H_2_O_2_. (a) The expression levels of AMPK and p-AMPK were detected by Western blot analysis. (b) The expression levels of Beclin1 and SQSTM1 were detected by Western blot analysis. (c) The expression level of LC3B-II/LC3B-I was detected by Western blot analysis. (d) The ROS levels were detected by DCFH-DA fluorescent probe (scale bar: 50 *μ*M). (e) Quantitative analysis of intracellular ROS level. (f) The expression levels of occludin and claudin-1 were detected by Western blot analysis. All data are presented as the mean ± SEM of three independent experiments. ^∗^*P* < 0.05, ^∗∗^*P* < 0.01, and ^∗∗∗^*P* < 0.001. SeNPs: selenium nanoparticles; H_2_O_2_: hydrogen; SQSYM1: sequestosome-1; LC3B: microtubule-associated protein light chain 3; ROS: reactive oxygen species.

As shown in Figures 10(a)–10(c), compared with the normal control group, the expression levels of p-AMPK, Beclin1, and LC3B-II/LC3B-I are significantly increased, and the expression level of SQSTM1 is significantly decreased in the H_2_O_2_ model group. However, SeNPs pretreatment inhibited the increase of p-AMPK, Beclin1, and LC3B-II/LC3B-I expression levels and the decrease of SQSTM1 expression level. Moreover, compared with the SeNPs + H_2_O_2_ group, the AICAR + SeNPs + H_2_O_2_ group showed a significantly reversed effect of SeNPs on the inhibition of p-AMPK, Beclin1, and LC3B-II/LC3B-I expression. In addition, compound C alleviated the increase of p-AMPK, Beclin1, and LC3B-II/LC3B-I expressions levels, and the decrease of SQSTM1 expression level induced by H_2_O_2_. These results suggested that SeNPs could regulate AMPK signaling pathway. As shown in Figures 10(d)–10(f), compared with the SeNPs + H_2_O_2_ group, AICAR + SeNPs + H_2_O_2_ group had a high level of ROS and a low levels of TJ proteins expression. However, compound C significantly reduced intracellular ROS level and increased TJs proteins levels in the H_2_O_2_ model group.

## 4. Discussion

The epithelial monolayer is the first line of defense in the gut. In addition to its role in nutrient absorption and metabolism, IEC also establishes a mucosal barrier that protects the body from pathogenic bacteria [29]. Transport of molecules in-between the intestinal epithelial cells is regulated through the presence of junctional complexes [30]. Mitochondria are the main sites for aerobic respiration in cells and are also the main organelles that generate ROS in cells [31]. Studies have shown that mitochondria are frequently damaged and are the main source of ROS during ERS [32]. Mitophagy is one of the mechanisms by which cells remove damaged mitochondria [33]. The formation of intestinal homeostasis depends on the function of ER for proper protein folding, modification, and secretion. Exogenous or endogenous risk factors can interfere with ER function and cause the intestinal barrier dysfunction, thereby activating the inflammatory response in the host. Homeostasis and repair of the gut are associated with autophagy and its regulatory mechanisms, maintaining gut barrier function in response to cellular stress by regulating TJs and protecting cells against apoptosis [34]. Recently, increasing studies have revealed that the pathogenesis of intestinal TJ barrier dysfunction is closely related to the induction of ERS and autophagy [35, 36]. Our previous research demonstrated that biogenic SeNPs by *Lactobacillus casei* ATCC 393 alleviated the increase of intestinal epithelial permeability, mitochondrial dysfunction, and mitophagy induced by oxidative stress [27]. However, the relationship of mitophagy-mediated the protective effects of SeNPs on oxidative stress-induced intestinal epithelial barrier dysfunction with ER stress remains unclear. In this study, the mitochondrial dysfunction is occurred in intestinal epithelial cells after oxidative stress and TM-induced ERS, as evidenced by the fact that the ROS, MMP, MPTP, ATP, and 8-OHdG levels are significantly increased, and accompanied by the occurrence of disruption of ER and mitochondrial ultrastructure in intestinal epithelial cells. In addition, our present study reveals that mitophagy is involved in the disruption of intestinal epithelial barrier after TM-induced ERS and the regulator effects of SeNPs.

In mammalian cells, ERS promotes UPR by activating the receptors located in the ER membrane [37]; there are three different ERS receptors in mammalian cells: IRE1*α*, PERK, and ATF6. Under normal circumstances, these receptor molecules interact with the chaperone protein GRP78 in the ER cavity in an inactive state; when ERS occurs, both IRE1*α* and PERK undergo oligomerization. It also undergoes autophosphorylation and functions through its downstream XBP-1 or eIF2*α* molecules [38, 39]. ERS causes the translocation of ATF6 from the ER to the Golgi apparatus. In the Golgi apparatus, ATF6 promotes the expression of a variety of ER chaperone proteins [40]. Under ERS conditions, UPR can not only promote cell survival but also induce cell apoptosis. Short-term mild and moderate ERS can cause phosphorylation and inactivation of eIF2*α* through PERK on the one hand, resulting in a decrease in the overall protein translation level, and a decrease in newly synthesized proteins entering the ER [41]; on the other hand, it can promote the expression of the ER chaperone protein GRP78 through ATF6 and XBP- and enhance the protein folding ability of the ER [42]. Persistent ERS in IECs may lead to impaired intestinal mucosal barrier function and further participate in the pathogenesis of ulcerative colitis [43]. Among the three signaling pathways of ERS, the PERK signaling pathway mainly regulates protein synthesis by recognizing and interacting with various unfolded proteins [44]. In this study, SeNPs markedly inhibited the expressions of p-PERK, XBP1, CHOP, and GRP78 in intestinal epithelial cells exposure to H_2_O_2_ or TM, indicating that the ERS is inhibited by SeNPs. These results suggested that the protective effects of SeNPs on the oxidative stress-associated intestinal barrier dysfunction may contribute to the inhibition of PERK-mediated ERS.

AMPK is involved in mammalian targets of rapamycin (mTOR)-mediated autophagy signaling, forming a more complex signaling cascade. AMPK is an intracellular energy state sensor that regulates cell survival under energy stress [45]. AMPK has been shown to inhibit the phosphorylation of mTOR, which is primarily responsible for protein synthesis, to promote cell proliferation and growth. Autophagy is suppressed by mTOR [46], a key metabolic regulator [47], and activated by the energy sensor-AMPK [48, 49]. During autophagy, LC3-I (the cytoplasmic form of LC3) is processed and recruited to autophagosomes to generate LC3-II, the lipidated form of LC3 [50]. Therefore, LC3-II is generally regarded as an important marker for detecting autophagic activity. LC3-II could induce mitophagy through the SQSTM1/p62 pathway [51]. Regulation of autophagy in IECs is linked to the complexity of multiple signaling pathways [52]. Selenium supplementation has also been reported to protect mitochondrial function, stimulate mitochondrial biogenesis, and alleviate oxidative stress during cerebral ischemia [53]. Likewise, selenium enhances mitochondrial respiration, increases mitochondrial content, and up-regulates mediators of mitochondrial biogenesis in placental cells and tissues [27]. Biogenic SeNPs can alleviate oxidative stress-induced mitochondrial dysfunction [54]. In this study, the autophagy of intestinal epithelial cells is significantly enhanced after oxidative stress and TM-induced ERS, as evidenced by the fact that the p-AMPK/AMPK, p-mTOR, LC3B-II/LC3B-I ratio, Beclin1, ULK1, PINK1, and Parkin are significantly increased, and that the expression of SQSTM1/p62 is decreased remarkably, accompanied by the occurrence of autophagosomes in intestinal epithelial cells. TM-induced ERS caused the activation and mitophagy-related AMPK signaling pathway and the disruption of intestinal epithelial barrier. However, SeNPs effectively attenuate the above phenomena. Furthermore, the PERK agonist (CCT020312) and the AMPK agonist (AICAR) reverse the protective effects of SeNPs on IPEC-J2 cells. The PERK inhibitor (GSK2656157) and the AMPK inhibitor (compound C) exhibit a similar effect on IPEC-J2 cells to that of SeNPs.

The reduced autophagy of IECs may be one of the players acting in the protective effects of SeNPs on the H_2_O_2_/TM-induced intestinal epithelial barrier disruption. Studies have found that the activity of the AKT/TSC/mTOR pathway is significantly reduced under ERS conditions, thereby promoting the occurrence of autophagy under ERS conditions [55]. In addition to the IRE1 pathway, the PERK-eIF2*α* pathway is also involved in the regulation of autophagy under ERS conditions. By promoting the expression of ATF4 and CHOP, the PERK-eIF2*α* pathway promotes the expression of a variety of ATG genes [56], thereby promoting the occurrence of autophagy under ERS conditions. Autophagy provides protection from a range of stressors including ERS [11, 12, 57], oxidative stress [22]. Disruption of this protection leads to increased intestinal susceptibility to damage and impaired intestinal regeneration [11, 12, 22, 57]. Under oxidative stress, energy deficiency, Ca^2+^ depletion, increased mRNA translation, and inflammatory stimulation, intracellular homeostasis is disrupted, and ERS is activated [58]. Several protein factors released under ERS conditions can directly induce the formation of autophagosomes and activate autophagy [59]. Autophagy plays two roles: On the one hand, moderate autophagy can maintain cell stability and survival; on the other hand, excessive autophagy leads to cell damage and even apoptosis [60] and aggravates UC [61]. ERS induces the conversion of LC3 from LC3-I to LC3-II and the formation of autophagosomes [62]. ERS can induce autophagy through multiple pathways. Moderate ERS can maintain intestinal homeostasis, while excessive ERS can induce intestinal inflammation by regulating ERS-susceptibility genes, inducing apoptosis of intestinal epithelial cells and damage to intestinal mucosal barrier function, and inducing the production of pro-inflammatory cytokines, which lead to the occurrence of UC [61]. Exploring the important mechanism of ERS-induced autophagy in the pathogenesis of IBD can contribute to the understanding of the pathogenesis of IBD and provide an effective method for the treatment of IBD in the future. The role of the ERS–autophagy signaling axis in the regulatory mechanism of SeNPs on intestinal epithelial barrier dysfunction needs further investigation.

## 5. Conclusion

In conclusion, oxidative stress induces the disruption of the intestinal TJ barrier and the increase of intestinal epithelial permeability and Ca^2+^ concentration in the cytoplasm, which is partly attributed to the enhanced ERS and mitophagy via the inhibition of ERS-related PERK/eIF2*α*/ATF4 and mitophagy-related AMPK/mTOR/PINK1 signaling pathway in intestinal epithelial cells. SeNPs effectively alleviated H_2_O_2_-induced intestinal epithelial barrier dysfunction by regulating the ERS and mitophagy. Our findings are helpful to elucidate the direct and incisive roles of ERS and mitophagy in the oxidative stress-induced disruption of intestinal epithelial barrier function and lighten a new direction to develop novel therapeutic strategies for intestinal barrier dysfunction induced by oxidative stress.

## Figures and Tables

**Table 1:** Sequences of oligonucleotide primers used for the mRNA levels analysis of endoplasmic reticulum-resident selenoproteins by real-time PCR.

Gene product^a^	Primer	
	Direction^b^	Sequence (5′ to 3′)
*β*-actin	F	GAGACCTTCAACACCCCAGCC
	R	AGACGCAGGATGGCATGGG
SELENOS	F	ACAGCCCTGCCAAGCAGAT
	R	AACAGGGAGGCTGGGTAACAC
SELENOF	F	GAGGCAGAGGCACCTGGAT
	R	CTGCTAAAGCCTCCTGTCGTTT
SELENON	F	CGGCTACATACCCCAGATGGA
	R	TGCTGCCACTTGATCTCCTC
SELENOK	F	ACATCTCGAACGGACAAGCG
	R	TGGCCCTCTTCCATCATCGT
SELENOM	F	CAGCTGAATCGCCTCAAAGAG
	R	GAGATGTTTCATGACCAGGTTGTG
SELENOT	F	GGCTTAATAATCGTTGGCAAAGA
	R	TGGCCCCATTGCCAGATA

^a^Selenoprotein F (SELENOF), selenoprotein N (SELENON), selenoprotein K (SELENOK), selenoprotein M (SELENOM), and selenoprotein T (SELENOT) resident in the endoplasmic reticulum, respectively. ^b^F: forward; R: reverse.

## Data Availability

The data will be made available after being requested from the corresponding author.
